# Inbreeding Implications on Genetic Diversity and Population Evolution of South American Brown Swiss Cattle

**DOI:** 10.1155/vmi/2405355

**Published:** 2026-05-23

**Authors:** Luis F. Cartuche-Macas, Miguel A. Gutierrez-Reinoso, Manuel Garcia-Herreros

**Affiliations:** ^1^ Veterinary Medicine Degree, Escuela Superior Politécnica Agropecuaria de Manabí Manuel Félix López (ESPAM), Calceta, 130602, Ecuador; ^2^ Veterinary Medicine Degree, Facultad de Ciencias Agropecuarias y Recursos Naturales, Universidad Técnica de Cotopaxi, (UTC), Latacunga, 050150, Ecuador; ^3^ National Institute for Agricultural and Veterinary Research (INIAV), Santarém, 2005-424, Portugal; ^4^ CIISA-AL4AnimalS, Faculty of Veterinay Medicine, University of Lisbon, Lisbon, 1300-477, Portugal, ulisboa.pt

**Keywords:** Brown Swiss, cattle, diversity loss, genetic variability, inbreeding

## Abstract

Inbreeding is a critical issue in both conservation and animal breeding, as it can severely impact survival, reproductive success and genetic diversity (GD) in domestic species. The Brown Swiss cattle were introduced in South America around a century ago; however, the breeding scheme–derived consequences have been neglected by breeders for numerous generations. The present research aimed to elucidate the population evolution, inbreeding and GD in introduced South American Brown Swiss cattle using genealogical databases. The study was based on official registered records from 8686 animals across multiple generations (until 2023). Different estimates related to population structure, including pedigree completeness, inbreeding rate (F), effective population size (Ne) and generation interval (GI), among others, were evaluated. Moreover, the characterisation of the probabilities of gene origin (effective number of founders [fe] and ancestors [fa]) and the GD loss were assessed. The results revealed that in the last period, GMax was 12.33, while the GCom and GEqu were 2.37 and 5.43, respectively. The mean GI was 7.11 years, and the mean F of 1.27% in the historical and the most recent populations was 5.60 and 2.78, respectively. The F increased between 0.10% and 0.50% per chronological period, showing an increasing tendency of the inbreeding values throughout time. The average Ne‐census, Ne‐ ΔF_p_ and Ne‐GEqu were 339.50, 143.80 and 95.40, respectively. Thus, a declining tendency of the effective population size was observed over time. The probabilities of gene‐origin–derived traits were 55 and 32 for fe and fa, respectively. The fe/fa ratio in the population was 1.72, indicating that occasional bottlenecks may have occurred in this breed. The GD analysis revealed a loss of 3.35% of total heterozygosity over time, though significant variability exists among chronological periods. In conclusion, this study revealed that considerable GD loss exists within the Brown Swiss cattle population, which needs better management mainly due to the relatively low founder effect and the low number of animals for maintaining a moderate reservoir of population diversity. Finally, these results emphasise the significance of overseeing GD and reducing inbreeding impacts. Successful breeding programmes are vital to improve conservation strategies and long‐term sustainable practices in South American Brown Swiss cattle.

## 1. Introduction

The Brown Swiss cattle were introduced in South America in the first half of the 20th century in order to improve other cattle breeds. This breed originated in the Swiss Alps and has great adaptability and longevity. Several breeds such as Allgäuer, Württemberger Braunvieh and Graubraunes Tiroler Gebirgsrind, as well as Swiss and Austrian brown cattle (Pinzgauer), were involved in its formation [1]. The Brown Swiss breed was introduced in Ecuador in the early 1940s, specifically in the highlands [2], and later in 1949 on the coast, as part of a crossbreeding plan with Brahman to increase milk production in this area [3].

It is worth mentioning that since about 10 years ago, the introduction of German Swiss and Austrian Braunvieh has taken place in Ecuador mainly in the form of artificial insemination doses. The main advantage of this breed is its great adaptability to different environments, especially in different production systems for dual‐purpose meat and milk production [4], and it is currently the second most important specialised breed in milk production in Ecuador. Thus, the monitoring of genetic diversity (GD) within cattle populations has become a routine activity within conservation and breeding programmes [5, 6]. The purpose of these analyses is to maintain diversity and avoid its loss due to factors such as the massive use of few predominant sires through assisted reproductive technologies (ARTs), such as artificial insemination carried out in different breeding centres from Europe (Germany, Switzerland and Austria) and North America (United States and Canada) [7, 8]. In Europe the breed has been mainly selected for traits such as milk quantity (kg) and milk quality (protein), as well as fitness and meat potential [9, 10], while in North America more importance is given to some production traits such as net merit, cheese merit, UDC, PPR and LPI [11, 12]. In any case, GD studies of this breed in some countries have shown that its GD has decreased over time [13] as well as the effective population size (Ne), as is the case in the German population [1] due to the common use of influential sires increasing the inbreeding rates [14, 15]. However, there are very few studies of inbreeding status and GD in this breed in South America. For example, Cartuche et al. [16] estimated the inbreeding levels of the Brown Swiss population born between 1953 and 2013; however, the GD was never determined.

The Ecuadorian Brown Swiss Association (EBSA) was created in 1951, from which the genealogical records and the genetic improvement of the breed were managed by the Ecuadorian government; however, from 2021 onwards, the Ministry of Agriculture and Livestock handed over the official management of the genealogical records to the EBSA [17]. It is worth mentioning that in the early 1970s, the application of the Agrarian Reform Law significantly reduced the number of breeders’ members [18] who decided to revitalise the association by importing live animals, embryos and artificial insemination doses from the United States and Canada. However, since then, insufficient attention has been paid to the possible consequences that this fact had on inbreeding and GD of the Brown Swiss population. In this regard, studies are currently necessary in developing countries on the basis of genealogical and genomic information [19, 20].

Therefore, the objective of this study was to evaluate the evolution of inbreeding and GD of the official registered Brown Swiss cattle population introduced in South America, specifically in Ecuador. The present study advances beyond previous work, particularly through the use of long‐term pedigree depth and a comprehensive set of diversity indicators. The parameters evaluated focused on demography, GD, probability of origin of genes and losses of GD. This will allow the implementation of analyses to maximise GD within the current population at the national level, which could be applied in other Central and South American countries where the same breed was introduced.

## 2. Materials and Methods

### 2.1. Ethical Statement

The present research did not require any animal handling, since the study was directly carried out using the records and databases provided by the Ecuadorian Brown Swiss Association (ABSE, Ecuador), the Council Dairy Cattle Breeding (CDCB, EEUU), the Braunvieh Schweiz (BRAUNVIEH) and the Canadian Dairy Network (CDN, Canada).

### 2.2. Pedigree and Population Structure

The genealogical database was provided by the EBSA. A total of 8686 registered animals were used, including 3317 bulls and 5369 cows, born until December 2023 (including genetic material from sires imported via AI doses). For the analysis, five populations were considered: the historical population (all individuals) and populations born from 1984 to 2023 taken at 10‐year intervals (1984–1993; 1994–2003; 2004–2013; 2014–2023) that included 8,686, 2,263, 2,721, 2,862 and 840 individuals, respectively. The sixth was the reference population, which was considered to be the one encompassing individuals with known sire and dam from the populations described above. Populations were defined because calculations related to GD, gene‐origin probabilities and founder analyses can only be performed by considering only individuals with both parents known or by comparing them with historical and current datasets as suggested by Casanovas Arias et al. [21] and Navas et al. [22]. ENDOG (v 4.8) software [23] was used to perform demographic and genetic analyses to quantify and trace GD back to ancestors.

The pedigree integrity index (PCI) was calculated following the assumptions of Navas et al. [22] from the first to the fifth generation and also the number of maximum generations (GMax), the number of complete generations (GCom) and the number of equivalent generations (GEqu) in the five defined populations. Moreover, the generation interval (GI) was calculated for the 4 gametic pathways from sire and dam to son and daughter, respectively, according to James [24]. For this purpose, the record of the birth date of each individual, together with that of its parents, was used. In parallel, gene flow between herds was evaluated according to the contribution of sires to the population [25].

### 2.3. Inbreeding and Coancestry

#### 2.3.1. Inbreeding Coefficient (F)

The F has been defined as the probability that two alleles taken at random are identical per offspring. The F was estimated using the algorithm proposed by Meuwissen & Luo [26], and the inbreeding increment (ΔF) per generation was calculated using the equation proposed by Gutiérrez et al. [23]:

(1) ΔF=Ft−Ft−11−Ft−1,

where *F*_*t*_ and *F*_*t*−1_: average inbreeding of the tth generation (*i* = 1, …, t).

Additionally, the inbreeding coefficient was estimated using the recursive method based on the algorithm proposed by Aguilar & Misztal [27], which assumes that the unknown parents do not have an inbreeding of zero but assumes that it is equivalent to the average inbreeding of parents born in the same year.

#### 2.3.2. Average Relatedness (AR)

Each individual’s AR coefficient was defined as the probability that two related individuals have inherited a particular allele of a single locus/gene from their common ancestor (this allele is known as IBD: identical by descent). AR was defined as the probability that a randomly selected allele from a population belongs to a specific individual, which was calculated using the vector *c*, where each element corresponds to the respective AR of an individual, defined by Gutiérrez & Goyache [28].

#### 2.3.3. Coancestry (C)

The C between two individuals is the probability that the genes, taken at random from each of the individuals, are IBD [29]. As a result, the C between two individuals is the F of their potential offspring. The C between two individuals is equal to the inbreeding coefficient of their offspring if the individuals are related [30]. It was also used to analyse the degree of relatedness and nonrandom mating, *α* within breeds. The C was estimated according to the algorithm of Colleau [31, 32].

#### 2.3.4. Nonrandom Mating (α)

The *α* was estimated as [30] the correlation of genes between two individuals in relation to the correlation of genes taken at random from the population (α), according to Caballero & Toro [33]. It indicates the degree of deviation from Hardy–Weinberg proportions and is related to the inbreeding coefficients according to Sheppard & Wright [34].

(2) 1−F=1−C1−α.

#### 2.3.5. Genetic Conservation Index (GCI)

The GCI was estimated from the genetic contribution of all founders, considering the proportion of genes from a founder animal in the pedigree under analysis according to Wang et al. [35]. The following equation was used:

(3) GCI=1∑pi2,

where ‘pi’ is the proportion of genes of founder ‘*i’* in the individual’s pedigree.

### 2.4. Pedigree Purging‐Based Measures

To analyse the effect of ancestry within different periods, pedigree purging methodologies were considered. Ballou’s coefficient of ancestral inbreeding (fa_Ballou) is one of the most widely used measures to detect pedigree‐based inbreeding purging [36, 37]. fa_Ballou is defined as the cumulative proportion of an individual’s genome that has been previously exposed to inbreeding in its ancestors [38], that is, the cumulative proportion that increases with each inbred ancestor in the pedigree. On the contrary, Kalinowski et al. [39] divided inbreeding, on the one hand, into ancestral (Fa_Kal), defined as those homozygous alleles that have already been found in previous generations, and additionally into new inbreeding (F_new), defined as where alleles are IBD for the first time. Considering that these inbreedings are estimated independently of the individual inbreeding coefficient, these values may be different. The value of Fa_Kal is zero if the traditional inbreeding (Meuwissen) is also zero, since there is a dependence on the existence of common ancestors in the maternal and paternal pedigree lines. The ancestral history coefficient (AHC) was also calculated. AHC was defined as the number of times that a random allele had been IBD during pedigree segregation [40]. Kalinowski’s inbreeding coefficients and the AHC were obtained by gene dropping, which was carried out using 100,000 replications. The GRAIN package Version 2.2 [40, 41] was used to calculate individual inbreeding coefficients based on the ancestral inbreeding, according to Ballou [38] and Kalinowski et al. [39], and an AHC as defined by Baumung et al. [40].

### 2.5. Effective Size (Ne)

Different methods can be used to compute the effective population size. In the present study the Ne‐census was obtained from the number of sires per generation (Sn) and the number of dams per generation (Dn). The Ne‐Fp value was obtained from the inbreeding coefficient of offspring (Ft) and the inbreeding coefficient of direct parents (Ft‐1) and *t*. Finally, the Ne‐GEqu was obtained from the sum of all known ancestors with (½)n and the individual inbreeding coefficient (Fi). The Ne calculation method was implemented in POPREP software.

### 2.6. Gene Origin Probabilities and Ancestral Contributions

#### 2.6.1. Number of Founders (f)

The *f* was defined as those individuals with unknown parents assumed to be unrelated and have an inbreeding coefficient of 0.

#### 2.6.2. Effective number of founders (fe)

The fe was defined as the number of founders that contribute equally and are expected to produce the same GD as the study population. It was estimated from the following equation [42]:

(4) fe=1∑k=1fqk2,

where ‘qk’ is the gene‐origin probability from ancestor ‘*k’* and ‘*f’* is the real number of founders.

#### 2.6.3. Effective Number of Ancestors (fa)

The fa was defined as the minimum number of ancestors that are not necessarily founders and that account for the full GD of a population, according to Boichard et al. [43]:

(5) fa=1∑k=1fpk2,

where ‘pk’ is the marginal contribution of an ancestor ‘*k’* that is not explained by other chosen ancestors and ‘*f’* is the real number of founders.

#### 2.6.4. Number of Founder Genome Equivalents (fg)

The fg was defined as the number of founders that would be expected to produce the same GD as the population under study if the founders were equally represented and no allele loss occurred. This was estimated from twice the inverse of the average C according to Caballero & Toro [33]. **Fe/fa and fg/fa** ratios were estimated to determine genetic bottlenecks and random genetic drift, respectively.

The genetic bottleneck was determined by calculating the number of ancestors in the population that contributed to 50% of the genes (fa50) and the ratio of fe/fa. The fa is expected to be smaller than the fe in the presence of a bottleneck, which can be indicated by the fe/fa ratio.

#### 2.6.5. Genetic Contributions

The genetic contributions were estimated for the top ten ancestors with the maximum genetic impact between 1999 and 2023. The marginal contribution of each major ancestor ‘j’ was calculated as proposed by Boichard et al. [43]. The CFC v.1.0 software was used to calculate ancestral contributions and gene‐origin probabilities [44].

### 2.7. GD

GD: The GD was estimated using the equation:

(6) GD=1−12fg.

GD‐loss: The population GD‐loss from the founder generation was estimated using 1 − GD. The GD‐loss due to unequal contribution of founders was estimated according to Caballero & Toro [33] using 1 − GD∗:

(7) GD∗=1−12fe.

The unequal contribution of founders relates to the fact that the genetic contributions of founders of specific populations may be of different proportions due to past directional mating (human‐mediated or not) during the process of population shaping. The difference between GD and GD∗ indicates the GD‐loss due to genetic drift accumulated from the population founding [42] and the effective number of nonfounders (Nenf).

### 2.8. Country Relationships

The relationships between countries were assessed through Nei’s minimum genetic distance [45] between subpopulations *i* and *j*, computed as described in Navas et al. [22] to evaluate intercountry relationships. Wright’s F‐statistics, or fixation indexes, which describe the statistically expected degree of heterozygosity reduction when compared to Hardy–Weinberg equilibrium (HWE) expectations, were computed following the principles outlined in Caballero & Toro [33]. The F‐statistics include F_IS_ (or F relative to the subpopulation), F_ST_ (the correlation between random gametes drawn from the subpopulation relative to the total population), and F_IT_ (F relative to the total population). F_IT_ can be decomposed into F_ST_, attributed to the Wahlund effect (a reduction in heterozygosity due to subpopulation structure), and F_IS_, resulting from inbreeding. Furthermore, self‐coancestry (*f*) was calculated as the probability that two alleles randomly selected from an individual (independently and with replacement) are IBD. Considering that the IBD condition can arise from either sampling the same allele twice or sampling two alleles that happen to be IBD, the coancestry of an individual with itself, denoted as f(A/A), is calculated as

(8) IBD=1+FA2,

where *F*(*A*) represents the inbreeding coefficient for that individual. It is crucial to recognise that coancestry in one generation translates into inbreeding in the subsequent one.

Subsequently, selfing, or autocoancestry, should be addressed. Selfing occurs when an individual mates with itself, a scenario often associated with hermaphroditism. In such cases, selfing equals the inbreeding coefficient (F) when autofecundation occurs. However, in the context of animal populations, the possibility of autofecundation is typically dismissed and considered to be 0, as is the case in our analysis.

### 2.9. Data Analysis and Software

The software used for the database analysis was ENDOG *v*. 4.8 [23], POPREP [46], CFC [44], INBUPGF90 [27], GRAIN package *v*. 2.2 [40, 41], DendroUPGMA [47] and MEGA [48], by means of which the demographic‐derived parameters, GD indices and gene‐origin probability were obtained.

## 3. Results

### 3.1. Pedigree Completeness‐Derived Parameters

**FIGURE 1:**
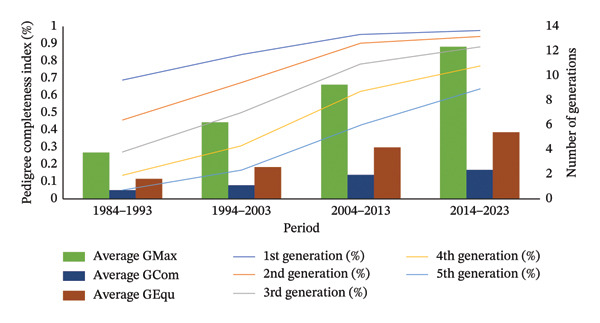
Pedigree completeness‐derived parameters in South American Brown Swiss cattle from 1984 to 2023. GMax: maximum number of generations; GenCom: complete number of generations; GEqu: equivalent number of generations.

Pedigree integrity is shown in Figure 1. A steady increase of the PC parameter was observed with values of approximately 12.33, 2.37 and 5.43 for the maximum, complete and equivalent number of generations, respectively. Finally, the pedigree integrity per generation reached in the last period higher values of 0.6 between the first and fourth generations.

### 3.2. GIs

The overall GI ranged from 7.36 to 5.6 years between the different periods, with a more pronounced reduction in the latter period (Table 1). When analysing the GI by the different genetic pathways, in the sire pathway, the GI was higher in the sire‐daughter pathway compared to the sire‐son pathway, while the dam‐son and dam‐daughter pathways had similar values. Finally, in the later period, there was a significant reduction in GI in all four pathways with values between 4.76 and 7.03 years.

**TABLE 1:** Generation interval (GI) of the four gametic pathways in introduced South American Brown Swiss cattle.

Period	Sire‐son	Sire‐daughter	Dam‐son	Dam‐daughter	Total
Historical	7.96 ± 0.13^aA^	8.31 ± 0.15^aA^	5.73 ± 0.11^bA^	5.85 ± 0.12^bA^	7.11 ± 0.05^cA^
1984–1993	8.96 ± 0.29^aA^	7.82 ± 0.29^aB^	6.30 ± 0.22^bB^	5.94 ± 0.23^bA^	7.20 ± 0.10^cA^
1994–2003	7.82 ± 0.19^aA^	8.48 ± 0.25^aA^	5.56 ± 0.17^bA^	6.04 ± 0.20^bA^	7.21 ± 0.08^cA^
2004–2013	8.10 ± 0.23^aA^	8.79 ± 0.29^aA^	6.06 ± 0.25^bAB^	5.89 ± 0.25^bA^	7.36 ± 0.08^cA^
2014–2023	5.04 ± 0.40^aB^	7.03 ± 0.46^bB^	3.95 ± 0.19^cC^	4.76 ± 0.29^acB^	5.60 ± 0.14^aB^

*Note:* Values are shown as mean ± SEM. Different superscript letters (a‐c) in a row represent significant differences among gametic pathways within the same period (*p* < 0.05). Different superscript letters (A‐C) in a column represent significant differences between periods within the same gametic pathway (*p* < 0.05).

### 3.3. Inbreeding, AR, Coancestry and Nonrandom Mating

**FIGURE 2:**
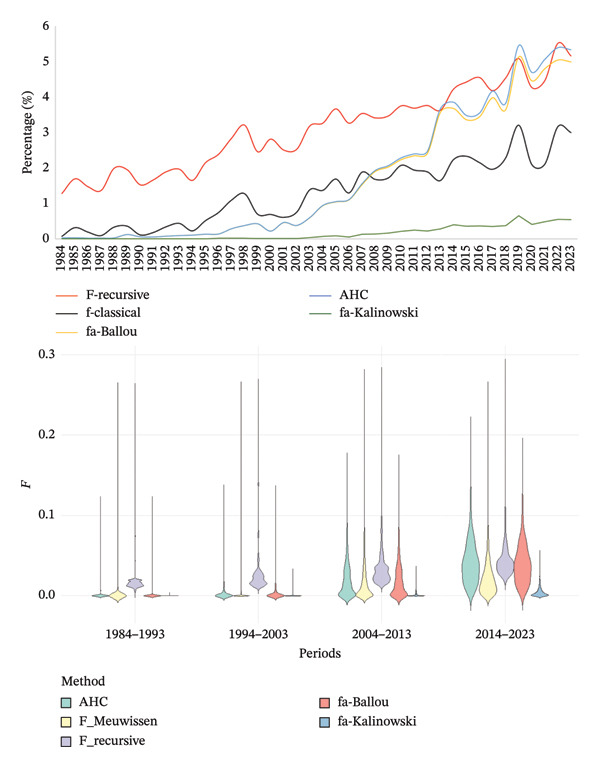
Trends of inbreeding coefficients estimated for South American Brown Swiss cattle from 1984 to 2023. Ancestral inbreeding per year (top graph) and per chronological period (bottom: violin plot) were calculated according to Ballou (Fa_Bal), Kalinowski (Fa_Kal), and Baumung (AHC: Ancestral History Coefficient). The individual inbreeding coefficient was calculated based on Meuwissen (F_Meuwissen), and the recursive method was used to calculate the recursive inbreeding (F_recursive).

Figure 2 shows the annual evolution of the inbreeding coefficients estimated by the different methods used. The recursive, AHC and Ballou methods remained with higher values compared to the classical method and Kalinowski, the latter being the one with the lowest values (Figure 2 top). Moreover, Figure 2 (bottom) shows the inbreeding measures in the evaluated periods. It was observed that the results obtained by the AHC and fa‐Balou methods were similar. The result obtained by Meuwissen’s method was slightly lower than that obtained by the recursive method. Finally, the result obtained by Kalinowski’s method remained at relatively low values.

### 3.4. GD Parameters Estimated Through Inbreeding

The GD parameters are detailed in Table 2. In this table it was observed that inbreeding value (F) increased from 0.28% to 2.78% between 1984 and 2023, with an increase of F of 0.50% in the last period. Likewise, it was determined that the population analysed in the last period is 92.38% related, with a GCI of around 5. Finally, the nonrandom mating (α) in the last two periods was positive.

**TABLE 2:** Inbreeding (*F*), average relatedness (AR), coancestry (C) and nonrandom mating (α) in South American Brown Swiss cattle.

Parameter	Historical (8,686)	1984–1993 (2263)	1994–2003 (2721)	2004–2013 (2862)	2014–2023 (840)
Inbreeding coefficient (*F*, %)	1.27	0.28	0.87	2.00	2.78
Inbreeding increment (Δ*F*, %)	0.31	0.10	0.26	0.46	0.50
Maximum inbreeding coefficient (%)	26.93	25	25.24	26.56	26.93
Inbred animals (%)	47.63	8.93	31.42	80.50	92.38
Highly inbred animals (%)	1.39	0.40	1.69	1.68	2.14
Coancestry coefficient (C, %)	0.92	0.50	0.88	1.20	1.27
Average relatedness (AR, %)	1.85	0.99	1.77	2.39	2.54
Genetic conservation index (GCI)	3.88	2.37	3.43	5.10	5.23
Nonrandom mating (*α*)	0.003542	−0.002237	−0.000183	0.008188	0.015349

#### 3.4.1. Ne

**FIGURE 3:**
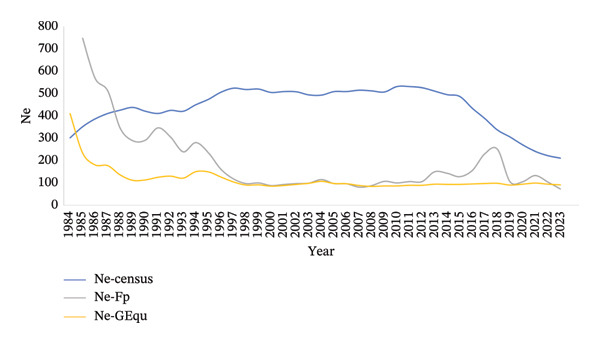
Evolution of the effective size (Ne) in introduced South American Brown Swiss cattle (1984–2023). Ne‐census: obtained from the number of sires per generation (S_n_) and number of dams per generation (D_n_); Ne‐Fp: obtained from the inbreeding coefficient of offspring (F_*t*_) and inbreeding coefficient of direct parents (F_*t-1*_) and *t*; Ne‐GEqu: obtained from the sum of all known ancestors with (½)^n^ and individual inbreeding coefficient (F_*i*_).

The effective population size (Ne) can be estimated by Coan, ΔF_p_, ΔF_g_, Δf_ln_, Ecg and census methods (Figure 3). The best methods based on the stratification test were Ne‐ΔFp, Ne‐Coan, Ne‐Ecg and Ne‐Cens. Estimates by these methods showed a decreasing trend. Finally, the Ne‐census estimated a value of 200 for the year 2023, while the Ne‐ΔF_p_ and Ne‐GEqu methods estimated a value of around 100 for the same year.

### 3.5. Gene Origin Probability, Ancestral Contributions and GD in Ecuadorian Brown Swiss Cattle

#### 3.5.1. Gene Origin Probability and Ancestral Contributions

The results of the gene‐origin probability analysis are shown in Table 3. There was a tendency to reduce the number of ancestors, founders and nonfounders at an accelerated rate in the last period. Moreover, the ratios of fe/fa and fg/fe for the last period were 1.72 and 0.27, respectively, which differed significantly from the previous period. The number of ancestors explaining 25% and 50% of the GD decreased between the first and the last period from 7 and 21 to 4 and 11, respectively.

**TABLE 3:** Gene‐origin probability and ancestral contributions in introduced South American Brown Swiss cattle.

Gene‐origin/Ancestral contribution parameters	Historical	1984–1993	1994–2003	2004–2013	2014–2023
Historical population (n)	8686	2263	2721	2862	840
Reference population (n)	6580	1194	1953	2624	809
Base population (one or more unknown parents)	148	334	111	29	10
Current base population (one unknown parent = half founder)	1622	735	657	209	21
Number of founders contributing to the reference population (n)	1703	975	1067	1015	735
Number of ancestors contributing to the reference population (n)	1449	737	768	670	403
Effective number of nonfounders (Nenf)	48.09	91.71	52.15	31.42	19.54
Effective number of founders (fe)	73	103	69	60	55
Effective number of ancestors (fa)	51	73	49	38	32
Founder genome equivalents (fg)	30.15	50.72	30.88	21.41	14.93
fe/fa ratio (fa/fe)	1.43 (0.70)	1.41 (0.71)	1.41 (0.71)	1.58 (0.63)	1.72 (0.58)
fg/fe ratio	0.41	0.49	0.45	0.36	0.27
fg/fa ratio	0.59	0.69	0.63	0.56	0.47

*Number of ancestors to explain:*					
25% of gene pool	5	7	7	4	4
50% of gene pool	21	36	20	14	11
75% of gene pool	127	187	108	61	40
100% of gene pool	1449	737	768	670	403

As for the most contributing ancestors in the reference population (Table 4), it was observed that 11 ancestors accounted for 50% of the GD, including 7 males and 4 females. Three of the sires, TOP ACRES ELEGANT SIMON, VICTORY ACRES JUBILAT EMORY∗TM, NORVIC TELSTAR were the most demanded in the decades 1980–1990 (21.25% of the GD), and two other sires (R N R PAYOFF BROOKINGS and SUN‐MADE VIGOR ET ∗TM) were in the decade 2000–2010, contributing to 5.81% of the GD. As for the females, IDYL WILD IMPROVER JINX was the one with the highest contribution to the GD, with 3.82%, since this female was a grand champion of the breed between 1982 and 1985, as well as TOP ACRES EJ WHIZZBANG, which is part of the great WHIZZBANG family, explaining 3.06% of the GD.

**TABLE 4:** Top ten ancestors with the greatest marginal contributions on the South American Brown Swiss cattle population.

Ranking	Name	YOB	Sex	Offspring (*n*)	Cumulative (%)	Individual (%)
1	TOP ACRES ELEGANT SIMON	1979	M	38	9.90	9.90
2	VICTORY ACRES JUBILAT EMORY∗TM	1984	M	126	17.02	7.12
3	R HART BC COLLECTION	1992	M	66	23.90	6.88
4	NORVIC TELSTAR	1976	M	66	28.13	4.23
5	IDYL WILD IMPROVER JINX	1978	F	9	31.95	3.82
6	HIGH SPRUCE STRETCHY EVE	1981	F	3	35.44	3.49
7	TOP ACRES EJ WHIZZBANG	1990	F	3	38.50	3.06
8	R N R PAYOFF BROOKINGS	2006	M	78	41.53	3.03
9	SUN‐MADE VIGOR ET ∗TM	2001	M	102	44.32	2.78
10	GENTLE BREEZE MAT CHRISTINE∗TW	1982	F	15	46.87	2.56

*Note:* M: male; F: female.

Abbreviation: YOB = year of birth.

#### 3.5.2. GD Loss

Table 5 shows the GD and its losses in each of the periods analysed. A downward trend in GD from 99.01% to 96.65% was observed between the first and the last period. The GD loss was highest in the last period (3.35%), of which 2.44% was due to genetic drift and 0.91% to the proportion of unequal contributions of the founders.

**TABLE 5:** Genetic diversity‐derived parameters in introduced South American Brown Swiss cattle.

Genetic diversity parameters	Historical	1984–1993	1994–2003	2004–2013	2014–2023
GD (%)	98.34	99.01	98.38	97.66	96.65
1‐GD (GD loss)	1.66	0.99	1.62	2.34	3.35
GD∗ (%)	99.32	99.51	99.28	99.17	99.09
Proportion of unequal contributions of the founders in GD loss (%)	0.68	0.49	0.72	0.83	0.91
Proportion of random genetic drift in GD loss (%)	0.97	0.50	0.89	1.50	2.44
Proportion of random genetic drift and bottlenecks in GD loss (%)	1.66	0.99	1.62	2.34	3.35

*Note:* The probability of gene origin is given by the effective number of founders; 1‐GD: Quantitative measure of the proportion of genetic diversity lost in a population compared to a base or founder population, often driven by inbreeding, genetic drift, or uneven founder contributions; GD∗: Genetic diversity in the reference population considered to compute the genetic diversity loss due to the unequal contribution of founders, effective number of founders (fe) and founder genome equivalents (fg).

Abbreviation: GD: genetic diversity.

### 3.6. Country Relationships

**FIGURE 4:**
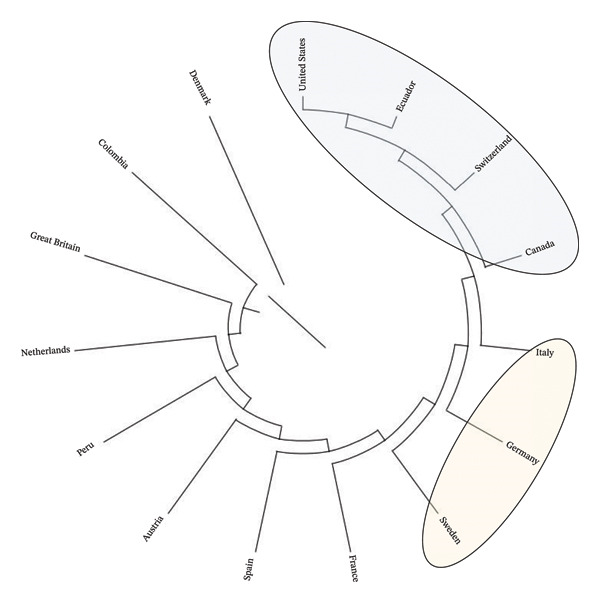
Dendrogram displaying countries after computing Nei’s genetic relationships of the Brown Swiss cattle. Dendrogram constructed based on Nei’s genetic distance (Wright′s and heterozygosity analysis) for the 15 subpopulations (countries) containing Brown Swiss cattle genetic resources. Clusters (ellipses) indicate the countries with more related Nei’s genetic distance showing the influence of genetic ancestors. Cluster 1 (United States, Ecuador, Switzerland and Canada); Cluster 2 (Italy, Germany and Sweden).

Table 6 shows Wright’s and heterozygosity statistics from the analysis of 15 subpopulations (Ecuador and origin of imported genetic material). The average inbreeding and co‐ancestry were 0.009808 and 0.008825, respectively. The average Nei genetic distance was 0.001539, and the average coancestry in the metapopulation was 0.007285. Regarding Wright’s parameter values, the FIS value was 0.000992, FST was 0.001551, and FIT was 0.002541. When evaluating the population structure at the country level, it was found that there are no nuclei but only multipliers, and in the case of Ecuador, 26.09% of national sires and 73.91% of foreign sires were used. The dendrogram (Figure 4) shows the genetic relationships between countries through the Nei distance. Ecuador is closely related to the United States in the first cluster within the first related group. In the same way, in the second cluster, the genetic relationship with Switzerland and Canada is observed.

**TABLE 6:** Wright’s fixation statistics and heterozygosity parameters when the subdivision criterion is the country of origin.

Parameters	Countries
Number of predefined subpopulations	15
FIS (inbreeding coefficient relative to the subpopulation)	0.000992
FST (correlation between random gametes drawn from the subpopulation relative to the total population)	0.001551
FIT (inbreeding coefficient relative to the total population)	0.002541
Mean inbreeding within subpopulations	0.009808
Mean number of animals per subpopulation	756.20
Average Nei genetic distance	0.001539
Mean coancestry within subpopulations	0.008825
Self‐coancestry	0.504904
Mean coancestry in the metapopulation	0.007285

## 4. Discussion

In the present study, the demography, inbreeding and GD loss were analysed over time from 1984 to 2023 in the Brown Swiss cattle introduced in Ecuador. This kind of study is crucial to avoid a greater risk of GD loss in the Brown Swiss cattle due to the influence of a few sires on the gene pool, which may increase inbreeding. Thus, diversified reproductive management is critical to avoid severe future bottleneck events. To address this issue, it is essential to adopt selection strategies that emphasise the inclusion of under‐represented genetic lines of sires or dams and prevent the excessive use of breeding stock, particularly via artificial insemination. Moreover, joint conservation initiatives with other groups may aid in restoring lost diversity and minimising risks. The Brown Swiss breed is geographically widespread in the three regions of Ecuador (highlands, coast and Amazon). This is due to the ease with which it has adapted to different environmental conditions, such as altitude, high and low temperatures, grazing production systems, dry and excessively humid climatic conditions, among others. Similar adaptations have been observed in the same breed in other countries, such as Ukraine [49]. Regarding the pedigree completeness‐derived parameters, the number of generations observed was 9 and 18 from the first to the last period analysed, which result was slightly lower than Brown Swiss populations analysed by Worede [19] in countries such as Austria, France, Italy, Germany, Slovenia, Switzerland and the United States, among others, which was around 23 generations. This fact would indicate that the Brown Swiss in Ecuador has a deep pedigree given that this breed has genealogical records from 1951 to the present. The PCI in the last period was 88.21% and 97.56% in the third and first generation, respectively, slightly higher than the breeds raised in Canada, such as Ayrshire (93%), Brown Swiss (80%), Canadienne (91%), Guernsey (97%) and Milking Shorthorn (75%) [50], as well as the Brown Swiss populations within INTERBULL analysed by Worede [15], which were around 57%–80%. Furthermore, it was similar to Swiss bovine populations (average of 5 generations) such as Brown Swiss (99.3%), Braunvieh (99.5%), Original Braunvieh (99.2%), Holstein (99.10%), Red Holstein (99.30%), Swiss Fleckvieh (99.20%) and Simmental (99.1%) [51]. Lower values were found in the Brazilian Holstein breed (17.42% and 78.02%) [52]. Since PCI values higher than 80% from the 3rd generation onwards guarantee reliability in GD estimators [53], it can be considered that the Brown Swiss breed from Ecuador has reliable information. A finding observed in the present study was the large difference between complete and maximum generations, which could indicate that there was a loss of genealogical information between individuals in the pedigree, as observed in the Tabapua and Nelore breeds in Brazil [54]. Furthermore, this fact indicates that both maximum and complete generations were increasing over time, which is an indicator of the increasing pedigree quality of the Brown Swiss breed in Ecuador. The equivalent generations observed (3.13 and 5.43, considering all periods) showed lower values than the Canadian breeds analysed by Melka, Stachowicz et al. [50] that showed values between 6 and 9 and higher than the Brown Swiss populations within Interbull [15] that showed values of 3.3 for Austria, 3.18 for Switzerland, 3.37 for Germany, 3.17 for France, 3.48 for Italy, 2.86 for Slovenia and 2.73 for the United States. These values obtained are due to the fact that the international genetic evaluations performed at Interbull focus mainly on Brown Swiss sires from Germany with a value of 8.04 in 2014 [1]. When comparing equivalent generations with other breeds, this value was also lower than that obtained in European red breeds with values between 8.72 and 9.24 [55]. In the Brown Swiss breed from Ecuador, the value of equivalent generations allows for estimating reliable parameters of GD. With regard to GIs, the value obtained in the Ecuadorian Brown Swiss had a tendency to reduce, being on average 5.59 years in the last period, as opposed to the previous period, which showed a value of 7.35 years. This effect is possibly due to the fact that breeders are using genomic‐selected sires, as has happened in other countries and breeds, for example, in Canada and the United States, the Holstein, Jersey and Brown Swiss breeds showed a tendency to reduce the GI since 2010–2011, especially in the sire‐son route. This is partly due to the onset and development of the genomic era, in contrast to the Ayrshire (USA‐CAN) and Guernsey (CAN) breeds [56, 57]. Despite a decrease in the GI, these values are not comparable with GI values obtained in other countries. For example, in dairy breeds in Canada, intervals between 4.21 and 5.75 years were obtained [50], values similar to the Brown Swiss International (∼6.90 years), although in this case only the paternal route was considered, which overestimated this variable [19], while in Ecuador a determining factor in obtaining this value could be due to the use of genomic‐tested sires (older than nongenomic‐tested), not taking advantage of the genomic technology itself. Thus, the GI value obtained in Ecuador (5.59 years) was similar to other specialised breeds in Ibero‐America, such as the Brazilian Holstein with 6.30 years [52]. In the sire‐daughter pathway, the Brown Swiss GI decreased by 3.05 years in the last periods, compared to the same breed in the United States between 2009 and 2016, which decreased by 3.9 years [57], although in 2022 a slight increase was observed, possibly due to the use of older individuals. In the sire‐daughter path, the GI showed a value from 8.79 years to 7.03 years in the last two periods, while in the United States, it decreased from 6.8 years to 3.9 years between 2009 and 2019 [57]. Although in both cases there was a decrease, in Ecuador it was not as strong, although since 2020 there has been a slight increase, possibly due to the use of proven sires, even if they were evaluated early with genomic technology. In the dam‐son pathway, the GI value showed a value from 6.06 to 3.95 years. Traditionally in the dam‐son pathway, cows are mated between the second and third calving as dams for sire production, reaching GI values higher than 6 years [58], this being the justification that in the period 2004–2013 the GI was around 6 years, while the reduction in the last period was due to sires/dams being selected between the first and second calving. In the dam‐daughter pathway, the GI value decreased from 5.89 to 4.76 years in the last two periods, possibly because the dams of daughters selected for replacement were found between the first and second calvings, similar to what happened in the Holstein, Jersey, Brown Swiss and Ayrshire populations of Canada [56].

Inbreeding analysis using various methods determined that the F‐Recursive, AHC and Fa_Ballou methods showed the highest values, ranging from 3.5% to 5% in the last period. The lowest values were for the fa_Kalinowski method, which showed values below 1%. Also, the traditional Meuwissen method showed intermediate values between 1.5% and 3% in the last period. This effect of method estimation was similar to that obtained in the German Holstein [59], Irish Holstein [60] and German Angler and Red‐White double‐purpose breeds [61]. The F‐Recursive, AHC and Fa_Ballou methods showed similar results throughout the periods studied because the recursive method considers that unknown parents within the pedigree possess some degree of inbreeding, considering it to be equal to the average inbreeding year of birth of the known parents. This means that if the population is more closely related, the average number of parents will be higher than in a nonrelated population. Further, the AHC method indicates how many times during pedigree segregation (gene dropping) a randomly taken allele has been in IBD status [40]. Ballou’s method, however, indicates the cumulative proportion of an individual’s genome that has been previously exposed to inbreeding in its ancestors [37]. These methods could show similar values given the high inbreeding value shown by the population by year of birth; however, since these populations have overlapping breeding, the inbreeding may be lower. These results on the trend of the methods for estimating F were similar to those for the Brown Swiss breed in Germany [1].

The GD‐derived parameters were obtained using the ENDOG software, which uses the inbreeding estimation method of Meuwissen & Luo [26]. The estimated historical inbreeding was 1.27%, which was higher than the value reported by Cartuche et al. [16], which was 0.12% for the Brown Swiss population born between 1953 and 2013. This is possibly due to the fact that the database used included purebred individuals and different degrees of crossbreeding, while in the present study only purebred individuals were included and also individuals born until 2023. Furthermore, the inbreeding estimated by the recursive method for the last period (2014–2023) was 4.54% (i.e., 1.64 times more than by the tabular method). This is because the recursive method considers that unknown parents have an inbreeding equal to the average of known parents born in the same year, according to Aguilar & Misztal [27]. In addition, this increase may also be due to the fact that the percentage of inbred individuals in the last period reached 92.38%. Thus, the trend of the inbreeding value in this breed increased from 0.28% to 2.78% between the first period (1984–1993) and the last period (2014–2023), as well as the AR value increased from 0.99% to 2.54%, respectively. Similarly, the percentage of inbred individuals increased to 92.38%. Inbreeding in the latter period was similar to that published by Worede et al. [15] for the international Brown Swiss population (*F* = 2.89, AR = 2.26) during the period 2000–2004. Regarding the inbreeding increment (Δ*F*), it was 0.50% between 2014 and 2023, which value was similar to the German Brown Swiss population showing Δ*F* = 0.36%–0.53% between 1990 and 2014 [1]. Finally, the GCI value was 5.23 in the last period (2014–2023), which value was higher than in other cattle breeds in Ecuador such as Holstein Friesian (1.42 between 2010 and 2021) and Montbeliarde (3.12 between 2019 and 2023) [62, 63] but less than the Charolais cattle population (7.18 between 2018 and 2022) [64]. The Ne value was calculated using the individual inbreeding rate per generation and is, therefore, less susceptible to overestimation due to low pedigree depth. Ne is also a parameter used to assess the endangered status of a population. According to the FAO [65], populations should have a minimum Ne value of 50–100 individuals to avoid possible increases in inbreeding, although to be sustainable in the long term, at least a value of Ne = 500 is necessary. In the present study, the stable trend of the Ne value in the Ecuadorian Brown Swiss was shown. The Ne value showed three trends. The first one until 1995, whose Ne value tended to increase up to 150 individuals, was followed by a second trend until 2004 with a value around 100. From 2004 onwards, it showed a slight increase with a value around 120. These values were higher than those estimated for the same breed in countries such as Canada (Ne = 47–76) [44, 50], France (Ne = 70–98), Switzerland (Ne = 70.6) [14] and an international Brown Swiss population (Ne = 63–204; 1995–2004), although they were similar to the German Brown Swiss population (Ne = 111.7) [1]. It is important to highlight the need for constant monitoring of the Brown Swiss population in Ecuador as well as the implementation of strategies to avoid the increase of inbreeding, such as mating planning considering F, C and AR; use of a higher number of sires; and use of genomics (ROH) for the selection of breeders. Regarding the gene‐origin probability and ancestral contributions, the fe values were higher compared to those obtained for fa in all periods, indicating that bottlenecks, genetic drift and contribution of certain lineages to subsequent generations have occurred over time. Similarly, this happened in dairy breeds in France [66], Montbeliarde in Ecuador [62] and dairy breeds in Canada [50]; however, other breeds did not have the same trend as occurred with the Sahiwal breed in Kenya [67] and the Brahman breed in Brazil [68]. Generally, the fe/fa ratio has been used to assess the effect of bottlenecks from the founders to the current population [43]. In the present study, the fe/fa ratio was increasing over time, reaching a value of 1.72 in the last period, a relatively low value compared to French dairy breeds that showed values between 2.73 and 3.90 [66], because they went through strong bottlenecks due to the indiscriminate use of few sires for artificial insemination in the 1990s. The same effect was also observed in this population that has suffered from pedigree bottlenecks, possibly because some lineages disappeared or there were factors that did not allow them to be passed on to subsequent generations. In beef breeds such as the Brazilian Brahman, a lower value (fe/fa = 1) has also been published [68] than the one obtained in the present study. Additionally, the fg/fe ratio was equal to 0.27, which indicates a significant GD loss possibly due to few founders contributing to the population, similar to other breeds in Ecuador such as the Charolais breed with an fg/fe = 0.22 [64], the Montbeliarde breed fg/fe = 0.13 [62], and the Holstein Friesian with an fg/fe = 0.05 [63]. These values indicate that the specialised breeds in Ecuador are going through a process of GD loss mainly due to the significant reduction of censuses in the genealogical register. This fact has also been described in Charolais and Limousin breeds in some European countries with fg/fe values between 0.09 and 0.20 [69]. Similarly, these values were relatively lower than Brahman, Nelore, Sindi and Tabapua breeds from Brazil with fg/fe = 0.41–0.71 [68, 70] and other European breeds such as Negra Andaluza fg/fe = 0.51 [71]. When analysing the fg/fa ratio, which evaluates the effect of genetic drift not related to the bottleneck, too‐low values were observed, indicating a high GD loss caused by genetic drift. In the case of Brown Swiss, the values found varied between 0.47 and 0.69, with a tendency to decrease over time. These values, compared to those obtained in European breeds such as Charolais and Limousin, were slightly lower, probably due to the massive and high importation of genetic material from French‐origin sires [69] in contrast to those from Ecuador, whose genetic material was mostly imported from the United States, Canada and Switzerland, which are genetically closely related [14, 15, 66]. The number of ancestors explaining 25% and 50% of GD were 4 and 11, respectively. The cumulative marginal contribution of the ancestors explaining 50% of the GD in the case of Ecuador was obtained from the sire VICTORY ACRES JUBILAT EMORY∗TM (BSUSA000000181329), which was the second sire that provided the highest GD (7.12%) compared to the Brown Swiss population from Germany, in which the same sire ranked tenth with 2.08% [1]. Also, the ancestors TOP ACRES ELEGANT SIMON and NORVIC TELSTAR were on the list of sires with more than 1000 daughters, with an expected future inbreeding of 7.2% and 6.9% [72]. Similarly, within the ancestry list there are 4 cows, of which IDYL WILD IMPROVER JINX represented 3.82% of the GD in the reference population. This cow was a reference within the breed, being champion of the 1985 World Dairy Expo, and of GENTLE BREEZE MAT CHRISTINE‐TW, a reference cow of the Brown Swiss breed with descendants that stand out today [73]. In this sense, it is shown that the influence of countries such as Canada and the United States has been marked in Ecuador with outstanding specimens of the Brown Swiss breed [73]. In this sense, it is shown that the influence of countries such as Canada and the United States has been marked in Ecuador with outstanding specimens of this breed.

The evolution of the GD in the Brown Swiss breed showed a GD loss over time similar to that observed in other breeds such as the Gyr breed in Brazil [74]. In general, the greatest GD losses (from 50.51% to 72.84%) occurred in all periods and were due to genetic drift. Moreover, the unequal contribution of founders was between 49.49% and 27.16%, similar to the Brown Swiss breed in Germany [1], Gyr in Brazil [74], Holstein, Montbeliarde and Charolais from Ecuador [62–64], being lower than Holstein, Jersey and Guernsey populations in Canada [44, 50, 75, 76]. GD loss due to genetic drift is common in cattle breeds, in particular in Ecuador, possibly due to factors such as the significant reduction of census registration in recent years (i.e., a reduction of population size) and, consequently, an increase of inbreeding and Ne values in the population. Together with the reduction of census registration, the lack of genomic‐derived information (genome‐related analysis) from Brown Swiss cattle individuals may be another limitation of the present study.

The use of genomic information for the GD analysis, and particularly inbreeding within populations, is becoming routine in populations with robust genetic improvement programmes and large population sizes that allow for reduced genotyping costs per individual [77]. This contrasts with developing countries such as Ecuador, where only genealogical information is available for monitoring these parameters. Although commercial genomic chips have been introduced in Ecuador in recent years, the costs remain prohibitive. This is compounded by the lack of knowledge among breeders about how to use this information, which they still find difficult to interpret and implement within their herds. Conceptually, the analysis of inbreeding through genealogical information, using IBD, may be less accurate than analysis using regions of homozygosity (ROH). ROH are contiguous segments of the genome where an individual inherits identical haplotypes from both parents, thus allowing for a broader analysis of GD and inbreeding. Possible ‘signatures of selection’ show patterns of shared ROH among individuals within a population, underlying genetic phenomena, recent common ancestry, or selection pressures that lead to greater homozygosity in particular genomic regions, as well as deciphering population histories, identifying regions under selection, and guiding animal genetics improvement strategies [78]. In Ecuador, until the Brown Swiss Association of Ecuador integrates genomic information into its programme or develops a genetic improvement programme, the only way to monitor inbreeding is through genealogical information.

Additionally, this diversity loss showed a positive relationship with decreasing fe, fa and fg, similar to those reported in German sheep breeds [79]. In the case of the German Brown Swiss population, the proportion of GD loss due to genetic drift was 68%, similar to the present study, possibly due to the effect of the use of sires of various origins, especially from Europe, such as ‘original Braunvieh‐OB’, Braunvieh‐BV and other sires with American Brown Swiss and Canadian‐BS blood [1]. These results indicate the need to implement measures to preserve GD since this breed is the second most important in milk production and is present in all three regions of Ecuador due to its great adaptability. Among these measures could be the creation of a semen germplasm bank considering pedigree and other genetic values, as well as the development of strategies to reduce F and increase Ne values as recommended by different authors [50, 66].

In the present study, the FIS value indicated that there was no inbreeding within the subpopulation, and the FST value indicated that there was a low level of genetic differentiation at the subpopulation and total population levels; however, the FIT value determined that the population had a low level of inbreeding in the total population. Thus, the FIS value obtained in the present study indicated an inbreeding level similar to the Negra Andaluza breeds, with a FIS value of 0.039 [71]; however, this value was different in the Braford breed in Argentina with a FIS = −0.0003 [80], the Sahiwal breed in Kenya with an FIS = −0.0071 [67], Charolais in Ecuador with FIS = −0.002614 [81] and FIS = −0.031 for the Gyr breed, with slightly negative values indicating a small excess of heterozygotes. The FST value in the present study indicated a very low level of genetic differentiation between the countries evaluated. This is possibly due to the great gene flow, mainly from the United States and Canada, although in recent years there has been an introduction of genetic material from Europe (although there is a high incidence of U.S. genetic material in these countries as well), such as Brown Swiss from Germany and Switzerland [1, 51, 82]. These values were slightly greater for the Sahiwal breeds in Kenya, with an FST = 0.0036 [67] and FST = 0.050 for the Gyr breed in Brazil [83] and greater than the Charolais breed in Ecuador, with an FST = 0.000610 [81]. In addition, a close relationship with the United States, Canada, Switzerland, Germany and Italy was verified in the dendrogram. Finally, the FIT value (0.002541) indicated a very low value of inbreeding in the total population compared to the Sahiwal breed from Kenya [67], the Charolais breed from Ecuador [79] and the Gyr from Brazil [81], which showed negative values (i.e., an excess of heterozygotes).

## 5. Conclusions

In summary, the low Ne value, the increased F coefficient over time, the completeness of pedigree (especially based on paternal pedigree), fe and fa values, ancestor contributions, and AR coefficient all show a decrease in GD in Ecuadorian Brown Swiss cattle. This may warrant consideration of improved management practices, given the unequal founder contributions and relatively small population size, which could affect the preservation of GD. Approaches such as introducing new sires with low AR coefficients and applying optimised mating strategies could potentially contribute to maintaining or enhancing GD, although their effects were not assessed in this study. Similarly, shortening the GI might help to accelerate genetic improvement and influence Ne, but this would require further evaluation to confirm its effectiveness in this population. The documented increase in inbreeding and reduction in genetic variability, alongside the influences of pedigree structure and founder representation, highlights the challenges faced in maintaining GD. To address these issues, improved management strategies, supported by the future integration of genomic tools, can play a crucial role in helping farmers and breeders mitigate inbreeding problems. As signs of a bottleneck may be emerging in some gene‐origin estimates, these combined efforts will be essential in preserving GD and ensuring the long‐term sustainability of Brown Swiss cattle breeding programmes.

## Author Contributions

Luis F. Cartuche‐Macas and Manuel Garcia‐Herreros contributed to the conception, design of study and acquisition of data. Luis F. Cartuche‐Macas and Manuel Garcia‐Herreros played roles in analysis and/or interpretation of data. Luis F. Cartuche‐Macas, Miguel A. Gutiérrez‐Reinoso and Manuel Garcia‐Herreros: drafting the manuscript. Luis F. Cartuche‐Macas, Miguel A. Gutiérrez‐Reinoso and Manuel Garcia‐Herreros: Critical review/revision.

## Funding

This research was funded by the Asociación Brown Swiss del Ecuador (ABSE) with the grant number ABSE‐PRINV‐2024–001 (Quito, Ecuador).

## Disclosure

All claims expressed in this article are solely those of the authors and do not necessarily represent those of their affiliated organisations, or those of the publisher, the editors and the reviewers. Any product that may be evaluated in this article, or claim that may be made by its manufacturer, is not guaranteed or endorsed by the publisher. All authors have thoroughly read and approved the final manuscript.

## Ethics Statement

Ethical review and approval were not required for the study because only previous data records were used from the research database, and none of the animals were handled or restricted at any time for this study.

## Conflicts of Interest

The authors declare no conflicts of interest.

## Data Availability

The data that support the findings of this study are available from the corresponding author upon reasonable request.
